# Effective Application of Improved Profit-Mining Algorithm for the Interday Trading Model

**DOI:** 10.1155/2014/874825

**Published:** 2014-01-29

**Authors:** Yu-Lung Hsieh, Don-Lin Yang, Jungpin Wu

**Affiliations:** ^1^Department of Information Engineering and Computer Science, Feng Chia University, No. 100, Wenhwa Road, Taichung 40724, Taiwan; ^2^Department of Statistics, Feng Chia University, No. 100, Wenhwa Road, Taichung 40724, Taiwan

## Abstract

Many real world applications of association rule mining from large databases help users make better decisions. However, they do not work well in financial markets at this time. In addition to a high profit, an investor also looks for a low risk trading with a better rate of winning. The traditional approach of using minimum confidence and support thresholds needs to be changed. Based on an interday model of trading, we proposed effective profit-mining algorithms which provide investors with profit rules including information about profit, risk, and winning rate. Since profit-mining in the financial market is still in its infant stage, it is important to detail the inner working of mining algorithms and illustrate the best way to apply them. In this paper we go into details of our improved profit-mining algorithm and showcase effective applications with experiments using real world trading data. The results show that our approach is practical and effective with good performance for various datasets.

## 1. Introduction

Data mining of Association Rules (AR) comes from knowledge discovery in large databases [1]. Using the application of AR on the stock market as an example, one can benefit from the rule: when the price of one stock goes up, the price of another stock goes up with 70% confidence at the same day. However, this rule cannot depict the information of when to buy or when to sell a stock. This form of AR only presents a fact that the prices of both stocks go up together. The investors do not know when to buy or when to sell the stock. To solve this problem in the financial market, Lu et al. [2] proposed the intertransaction mining (ITM) of association rules.

The ITM is very popular for investors to find rules like “when the price of one stock goes up, then the price of another stock will go up the next day with 70% of confidence.” This rule indicates that one may win 7 out of 10 times. With ITM we still cannot be sure to have a profit as illustrated in the next example. Assume that an investor gets 1 dollar for each win and loses 3 dollars otherwise. Based on a simple calculation, we get a loss of 2 dollars from 10 trades, which is 7 × 1 − 3 × 3 = −2 dollars. This example amplifies the point that high confidence does not necessarily mean high profit.

In the investment market, although every trader wants to make a profit, taking a loss is almost inevitable and becomes a risk. In particular in the futures market, the main purpose is to allow those who wish to manage price risk to transfer that risk to those who are willing to take that risk in return for an opportunity to profit. In addition to the profit and risk, what are concerned by investors the most? For example, if two trading results have the same profit and risk with two different winning chances of 1% and 99%, then the investor would prefer the latter over the former. Most investors have low psychological endurance to bad performance of their stocks and prefer the trading with a higher winning chance. Here the winning chance is called WinRate. Therefore, the combination of *high profit*, *low risk,* and *high WinRate* becomes the choice of the most investors.

Profit-mining (PM) [3] is such an alternative solution to ITM that better meets the requirements of investors in financial market. PM considers more of investor's expectation on mined results which include profit, risk, and WinRate. The resultant profit rule has the form “Buy stock *XYZ* when an event (or a signal) *A* occurs and sell stock *XYZ* when an event (or a signal) *B* occurs; then you have profit = *P*, risk = *R*, and WinRate = *W*%.”

The concept of PM is similar to utility mining from [4]. However, Chan et al. use a sales manager's perspective to mine profitable result that is important to the customer. Traditional statistical correlations may not measure how useful an itemset is in accordance with customer preferences. Although utility mining solves the problem from a sales manager's perspective, the concerns from customers in financial market have not been addressed. PM can be used to solve the problem. The utility mining uses a fixed weight to compute the profit while PM uses a dynamic approach depending on two trading orders. PM can find trading rules and trading results based on the trading model and investor's expectations. To find the profitable rules more efficiently, we improve our PRMiner algorithm [5] and illustrate its usage with real world applications in this paper.

The rest of the paper is organized as follows.  Section 2 presents the related work and Section 3 introduces problem definitions and reviews our research approach. The improved PRMiner algorithm, profit rule mining examples, and real world applications are described in Section 4 while Section 5 discusses experimental results.  Section 6 concludes the paper and describes our future study.

**Pseudocode 1:**
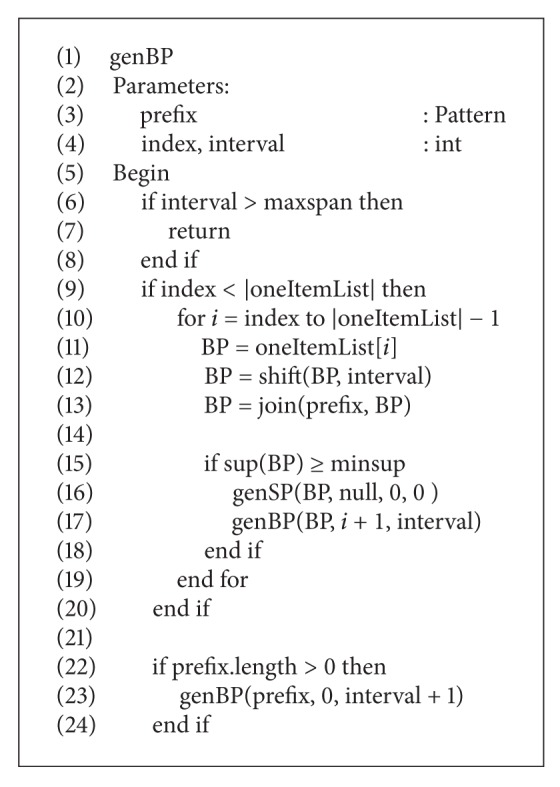
The pseudocode of genBP.

**Pseudocode 2:**
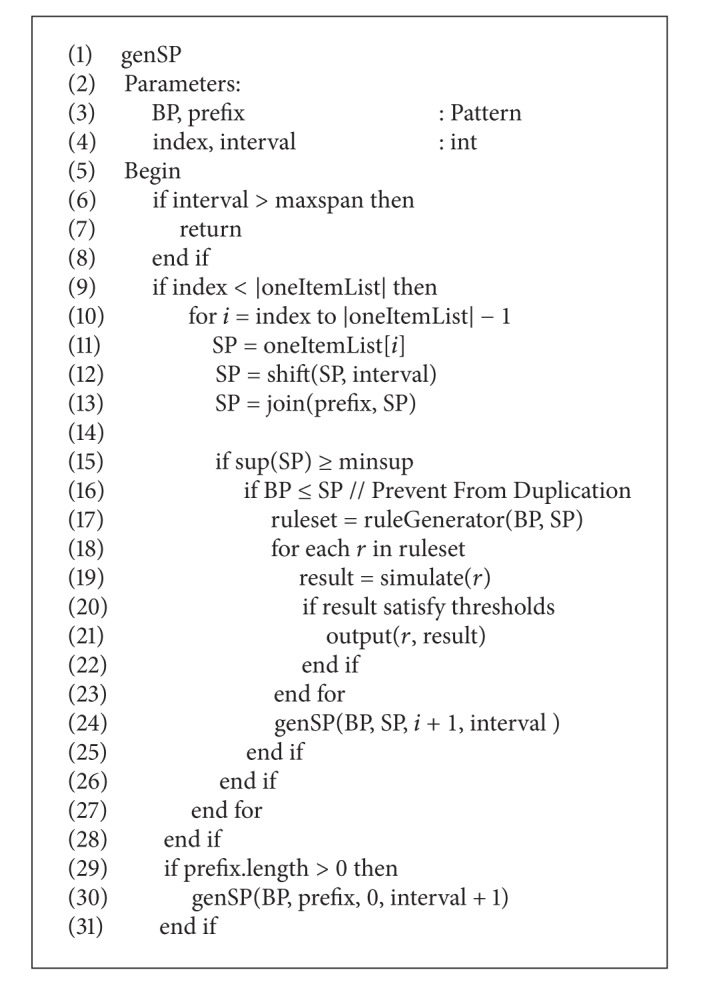
The pseudocode of genSP.

## 2. Related Work

Agrawal et al. [1] proposed the association rule mining for finding the rule of related itemsets with high frequency and confidence from transactional databases. Lu et al. [2] presented the intertransaction association rules mining to predict if some stock prices will go up or down based on the previous performance of other stocks. Chan et al. [4] developed an objective-directed utility mining to find the top-K high utility closed patterns supporting a given business objective. Su and Huang [6] used model free estimators to predict the price of stock. Magdon-Ismail and Atiya [7] presented their research on risk management in financial market, which is critical for investors. Zhang and Zhou [8] revealed the fact that “data mining in finance is involved with the features of data, target applications, and domain models leading to a conceptual framework consisting of these three dimensions.”

Boetticher et al. [9] performed several studies to mine the financial data in which the result is presented with profit but lacks risk information. Hsieh et al. [10] used the ITM to mine stock data and to study upstream and downstream causal relationship in stock market. In real world applications, Trade Station [11] can be used to build program models that can perform trading simulation. Based on the simulation, Trade Station generates reports to show the expected profit, risk, and other trading results. Recently, Bodas-Sagi et al. [12] proposed an evolutionary approach to optimize the parameters of two important technical indicators (RSI and MACD) by considering the objectives of maximal profit and minimal risk. However, Hsieh and Yang [3] had already applied a profit-mining model to solve the problem using all three factors of profit, risk, and winning rate (i.e., WinRate). In this paper we make further improvements on the profit-mining algorithm PRMiner and its implementation [5]. Practical applications and experiments are used to show how well our improved algorithm can work on real world trading data.

## 3. Problem Definitions and Our Research Approach

In this section we describe the interday trading model [5], transactional database (TDB), trading rules, trading results, and profit rules. Using trading rules we can simulate trading in the interday model for the target TDB and generate trading results with profit, risk, and WinRate. The rules meeting users' expectation are profit rules.

### 3.1. Interday Trading Model

We define TC as a set of trading commands consisting of *Buy* and *Sell*. Let TO = {*tc*, *qty*, *price*} denote the form of a Trading Order where *tc* ∈ *TC*, *qty* ∈ *N* ^+^, and *price* ∈ *R* ^+^. With no loss of generality, we set the value of *qty* to 1. We say that a TO is either a Buy-Order (BO) if *tc* = “*Buy*” or a Sell-Order (SO) if *tc* = “*Sell.*”

Let MP be a set of market positions containing *None*, *Long,* and *Short*. We use {*mp*, *hqty*, *hprice*} to denote the form of a position POS where *mp* ∈ *MP*, *h* *qty* ∈ {0,1}, and *h*price ∈ *R* ^+^. We say that a POS is in a *close* position if *mp* = “*None*,” *hqty* = 0, and *hprice* = 0. Otherwise, a POS is either in a *long* position if *mp* = “*Long*” or in a *short* position if *mp* = “*Short.*” In addition, let HPOS denote a *hold* position representing a position of an investor in hold before or after trading. Here HPOS is also a kind of POS.

Therefore, the HPOS_0_ at time *t* = 0 is in a *close* position {*None*, *0*, *0*}. A *close* position means that an investor does not hold any stock. Therefore, it does not matter whether stock prices will go up or down. A *long* position means that an investor expects future stock prices to go up for a chance to make profit. On the other hand, a *short* position means that an investor expects to make profit in the future if the stock price goes down.

**Figure 1:**
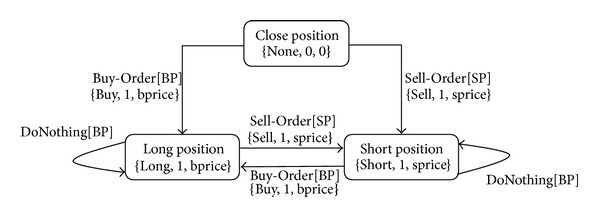
The state machine of an interday trading model.

We use a state machine in Figure 1 to explain the interday trading model [5] with three hold positions: *close*, *long*, and *short*. In Figure 1, a Buy-Order (BO) is generated when a Buy-Pattern (BP) appears in the transaction database (TDB) and a Sell-Order (SO) is generated when a Sell-Pattern (SP) occurs. The BP and SP are patterns to be mined. A Buy-Order or Sell-Order is used to change the status of a *hold* position (HPOS) at the beginning to other positions in Figure 1. At time *t*, a BO_*t*_ or SO_*t*_ is generated to change a *hold* position from HPOS_*t*−1_ to HPOS_*t*_. The action “change” is also called an offset. We define an operator “−” to indicate the operation of an offset. Thus, the operator “−” uses TO_*t*_ to offset a *hold* position from HPOS_*t*−1_ to HPOS_*t*_. The definition of the operator “−” among HPOS_*t*−1_, TO_*t*_, and HPOS_*t*_ is as follows:

(1) $$\begin{array}{c} \text{HPO}{\text{S}}_{t}=\text{T}{\text{O}}_{t}-\text{HPO}{\text{S}}_{t-1}. \end{array}$$

The results of the offset operation are defined below:

(2) HPOSt.mp=“Long,” if  TOt.mp=“Buy,”  t>0HPOSt.mp=“Short,” if  TOt.mp=“Sell,”  t>0HPOSt.hqty=TOt.qty, if  t>0HPOSt.hprice=TOt.price, if  t>0.

At time *t*, if there exists a trading order TO_*t*_ that changes HPOS_*t*−1_ to HPOS_*t*_, then we call its previous trading order TO_*t*−1_ a complete trading order (CTO). For example, the investor holds a *close* position HPOS_0_ = {*None*, 0, 0} as shown in top of Figure 1. When a BP occurs in the TDB at time *t* = 1 and a BO_1_ = {*Buy*, 1, *b* *price*_1_} is generated, it changes HPOS_0_ to HPOS_1_ by following the arrowhead line from “*Close* Position” to “*Long* Position.” HPOS_1_ = BO_1_ − HPOS_0_ = {*Buy*, 1, *b* *price*_1_} − {*None*, 0, 0} = {*Long*, 1, *b* *price*_1_}. Then the state does not change until a SP occurs in the TDB. DoNothing[BP] in Figure 1 means that no action will take place when a BP appears.

Assume that a Sell-Pattern SP occurs at time *t* = 2 and a Sell-Order SO_2_ = {*Sell*, 1, *s* *price*_2_} is generated to offset HPOS_1_ to become HPOS_2_ by following the arrowhead line from “*Long* Position” to “*Short* Position.” HPOS_2_ = SO_2_ − HPOS_1_ = {*Sell*, 1, *s* *price*_2_}−{*Long*, 1, *b* *price*_1_} = {*Short*, 1, *s* *price*_2_}. Then, BO_1_ is a complete trading order (CTO) because SO_2_ changes the state from HPOS_1_ to HPOS_2_. The remaining two offset cases from “*Close* Position” to “*Short* Position” and from “*Short* Position” to “*Long* Position” can be derived in a similar manner.

### 3.2. Transactional Databases

Let *E* = {*e*_1_, *e*_2_,…, *e*_*m*_} be a set of *m* items. An item represents an event or a trading signal. Let TDB be a transactional database which contains a set of *n* transactions {tr_1_, tr_2_, …, tr_*n*_}. Let (*TID*_*j*_, *ItemSet*_*j*_, *Price*_*j*_) denote the form of a transaction tr⁡_*j*_ where *TID*_*j*_ is a consecutive number and *TID*_*j*_ > 0, *ItemSet*_*j*_ contains a set of items and *ItemSet*_*j*_ ∈ *E*, and *Price*_*j*_ ∈ *R* ^+^.

Table 1 shows an example of TDB with three attributes TID, ItemSet, and Price as in [5]. For simplification, we ignore mega-transactions without itemset in their base transaction. TID is a transaction ID possessing the time feature to establish the trading sequence and identify the set of items and trading result in the fields of ItemSet and Price, respectively. The investors use various techniques and information to determine the best time to buy or sell stocks, leading to produce trading signals which are recorded in the database as itemsets. In Table 1, there are ten such transactions.

The definition of a pattern in PM is similar to that of ITM. Let a pattern be a set of items (or an itemset) in the form of {*e*_1_(0),…, *e*_*n*_(0),…, *e*_1_(1 − *i*),…, *e*_*n*_(1 − *i*),…, *e*_1_(1 − *w*),…, *e*_*n*_(1 − *w*)}, where *w* is a user specified variable called *maxspan*, *e*_*j*_ ∈ *E*, *j* = 1 ⋯ *n*, and 1 < *i* < *w*. The value *w* of *maxspan* denotes the maximal number of transactions in the TDB containing a pattern. We use a sliding window approach to define such a window of *w* transactions as a megatransaction. The last transaction containing an item or itemset in a megatransaction is called a *base transaction* and the rest of transactions are *extended transactions*. The number in the parenthesis of *e*_*j*_(*i*) is the relative position of an extended transaction with respect to the base transaction in a megatransaction. This number starts from zero for the base transaction. The first extended transaction above the base transaction is minus one, where a minus value indicates an opposite direction from the moving direction of sliding windows.

For example, assuming that *maxspan* = 2 in Table 1, there are six megatransactions *M*1, *M*2,…, *M*6. The megatransaction *M*1 contains two transactions with *TID* = 1 and *TID* = 2, *M*2 contains two transactions with *TID* = 2 and *TID* = 3, and so forth. The megatransaction *M*2 has five patterns *a*(0), *b*(0), *a*(0)*c*(−1), *b*(0)*c*(−1), and *a*(0)*b*(0)*c*(−1). The third pattern *a*(0)*c*(−1) implies that *a*(0) and *c*(−1) are in the same megatransaction *M*2. The same pattern occurs in the megatransaction *M*5 as well. The number of −1 in the parenthesis of *c*(−1) indicates that item *c* is in the first extended transaction of *TID* 2 above the base transaction of *TID* 3. Since the pattern *a*(0)*c*(−1) occurs at *TID* 3, the trading price 500 of pattern *a*(0)*c*(−1) is set at the base transaction of* TID* 3, not at *TID* 2.

### 3.3. Trading Rules

As described in our trading model of Figure 1, we use BP and SP patterns to determine the trading orders of Buy and Sell, respectively. If BP and SP occur at the same time causing a semantic ambiguity, an investor does not know what trading action (Buy or Sell) to take. A new attribute of trading - priority (*TP*) is required to solve the problem. Now we can define the format of a trading rule as {*TP*, *BP*, *SP*} where *TP* ∈{*BF*, *SF*}, “*BF*” stands for Buy-First, and “*SF*” stands for Sell-First. When a semantic ambiguity situation occurs at a market position *MP* = “*None*,” if *TP* = *BF*, then the generated trading order is a Buy-Order (BO). If *TP* = *SF*, the generated trading order is a Sell-Order (SO). For example, given a trading rule {*SF*, *a*(0), *b*(0)} with stock *XYZ*, the trading priority *SF* tells the investor to sell the stock *XYZ* when *BP * = *a*(0) and SP = *b*(0) occur simultaneously at a close position (i.e., *MP* = “*None*”). Then the investor can buy the stock *XYZ* when *BP * = *a*(0) occurs again. If *BP * = *a*(0) and *SP * = *b*(0) do not occur simultaneously, buy the stock *XYZ* when *BP * = *a*(0) occurs and sell the stock *XYZ* when *SP * = *b*(0) occurs.

### 3.4. Trading Results

Trading results are generated by using trading rules in a trading simulation. Our trading result includes *Profit*, *Risk*, and *WinRate*. In real world practice, each trading in a trading simulation must pay some trading fee and tax. In our trading model one must specify the handling* fee* for each trading to cover the fee and tax as paid in real world.

#### 3.4.1. Profit

Let *DB*_Begin_ and *DB*_End_ be two TIDs of the first and last transactions in a TDB, respectively. *P* is a function to get the price at a specific TID, denoted as *P*(*TID*). Equations (3) and (4) are used to compute the net profit (*NP*) of a trading rule. Consider the following:

(3) $$\begin{array}{c} NP_{i,j}=\{\begin{array}{cc} P\left(T_{j}\right)-P\left(T_{i}\right)-2\times fee & \text{if}\;\;MP_{i}=“\text{Long,”}\\ P\left(T_{i}\right)-P\left(T_{j}\right)-2\times fee & \text{if}\;\;MP_{i}=“\text{Short.”} \end{array} \end{array}$$

The net profit of a trading rule is to summarize all *NP*s. Consider the following:

(4) $$\begin{array}{c} \text{Profit}=\sum_{}^{}NP_{i,j}. \end{array}$$

**Figure 2:**
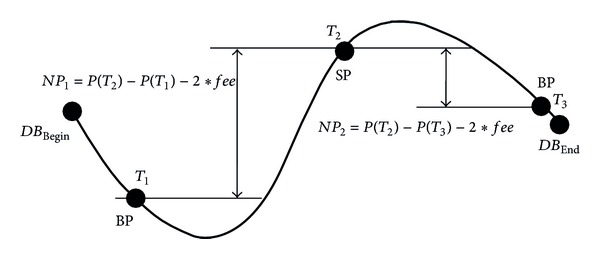
A stock price versus trading time curve for computing the profit of a trading rule.

To better explain the net profit calculation, we use a stock price versus trading time curve in Figure 2 [5] based on the TDB in Table 1. The *X*-axis is trading time and the *Y*-axis is stock price. We start from *DB*_Begin_, where HPOS_0_ = close position. The first pattern in Figure 2 is a BP which appears at transaction T_1_ and a Buy-Order BO_1_ = {*Buy*, 1, *P*(*T*_1_)} is generated to offset HPOS_0_ to HPOS_1_. The calculation is HPOS_1_ = BO_1_ −  HPOS_0_ = {*Buy*, 1, *P*(*T*_1_)} − {*None*, 0, 0} = {*Long*, 1, *P*(*T*_1_)}. At the *T*_1_ point, the investor holds a long position HPOS_1_ = {*Long*, 1, *P*(*T*_1_)} expecting a profit or a loss if the price of stock goes up or down in the future, respectively. The second pattern SP occurs at the *T*_2_ point and SO_2_ = {*Sell*, 1, *P*(*T*_2_)} is generated to offset HPOS_1_ to HPOS_2_. The calculation is HPOS_2_ = SO_2_ −  HPOS_1_ = {*Sell*, 1, *P*(*T*_2_)} − {*Long*, 1, *P*(*T*_1_)} = {*Short*, 1, *P*(*T*_2_)}. At *T*_2_, we get the first net profit *NP*_*1*_ = P(*T*_2_) − P(*T*_1_)−2 × *fee* because the value of *MP*_1_ is “*Long*.” At the *T*_2_ point, the investor holds a short position = {*Short*, 1, *P*(*T*_2_)} expecting a profit or a loss if the price of stock goes down or up in the future, respectively. The 3rd pattern BP occurs at the *T*_3_ point and BO_3_ = {*Buy*, 1, *P*(*T*_3_)} is generated to offset HPOS_2_ to HPOS_3_ = {*Long*, 1, *P*(*T*_3_)}. We have the second net profit *NP*_2_ = *P*(*T*_2_) −  *P*(*T*_3_) −  2 × *fee* because the value of *MP*_2_ is “*Short*.” Since the *T*_3_ point is next to the end, there is no trading pattern to expect. The profit of the trading rule is *NP*_1_ + *NP*_2_.

#### 3.4.2. Risk

We use the three variables *Consecutive Loss* (*CLoss*), *Draw Down* (*DD*), and *Run Up* (*RU*) to define the risk of a trading rule. Their initial values are zero and maximal values are initialized to zero. *CLoss* is used to record the consecutive loss of *net profit* (*NP*) by using the following equation:

(5) CLoss  at  time  t:  CLosst=CLoss(t−1)+NPt,where  CLoss0=0, CLosst=0if  (CLoss(t−1)  +  NPt)>0.

**Figure 3:**
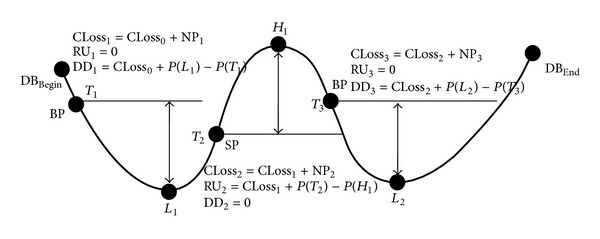
A stock price versus trading time curve for computing the risk of a trading rule.

DD and RU are used to record the risk during the trading process when *MP* is “*Long*” and “*Short,*” respectively. Let *T*_*i*_ and *T*_*j*_ be two TIDs where *T*_*i*_ < *T*_*j*_. Let min⁡*P*(*T*_*i*_, *T*_*j*_) and max⁡*P*(*T*_*i*_, *T*_*j*_) be two functions for getting the minimal and maximal prices, respectively, from *T*_*i*_ to *T*_*j*_, where *T*_*i*_ < *T*_*j*_ and *T*_*i*_, *T*_*j*_ ∈ *TID*. We use Figure 3 to show the calculation of *DD* and *RU*.

*DD* records the difference between the buying price and the lowest price where the difference must be less than 0; otherwise *DD* = 0. The location of the lowest price is between the buying point and the next selling point or between the buying point and *DB*_End_. Two examples are shown at *L*_1_ and *L*_2_ in Figure 3. The *L*_1_ point is the lowest point between *T*_1_ (where a BP occurs) and *T*_2_ (where a SP occurs) while the *L*_2_ point is the lowest point between *T*_3_ (where a BP occurs) and *DB*_End_. The equation to compute *DD* is shown below:

(6) $$\begin{array}{c} DD_{t-1}=C\text{Los}{\text{s}}_{t-2}+\min P\left(T_{t-1},T_{t}\right)-P\left(T_{t-1}\right)\\ \text{if}\;MP_{t-1}=“\text{Long”,}\;t>1,\;\;T_{t}\neq \text{Null} \end{array}$$

However, if the BP at *T*_*t*_ is next to the *DB*_End_, there is no SP to expect and (7) is used to compute *DD*. Otherwise, the value of *DD* is 0. Consider the following:

(7) $$\begin{array}{c} DD_{t}=C\text{Los}{\text{s}}_{t-1}+\min P\left(T_{t},DB_{\text{End}}\right)-P\left(T_{t}\right)\\ \text{if}\;MP_{\left(t-1\right)}=“\text{Long”,}\;t>0,\;\;T_{t}\neq DB_{\text{End}}. \end{array}$$

The definition of *RU* is similar to *DD*, but *RU* works at *MP* = “*Short*” and records the difference between the selling price and the highest price. The location of the highest price is between the selling point and the next buying point or between the selling point and *DB*_End_. One example is shown at the *H*_1_ point in Figure 3. The *H*_1_ point is the highest point between *T*_2_ (where a SP occurs) and *T*_3_ (where a BP occurs) such that the risk at *T*_2_ is *P*(*T*_2_) −  *P*(*H*_1_) since *MP*_2_ = “*Short.*” The following two equations (8) and (9) corresponding to equations (6) and (7) are used for *RU*, respectively:

(8) $$\begin{array}{c} RU_{t-1}=C\text{Los}{\text{s}}_{t-2}+P\left(T_{t-1}\right)-\max P\left(T_{t-1},T_{t}\right)\\ \text{if}\;MP_{t-1}=“\text{Short”,}\;t>1,\;\;T_{t}\neq \text{Null,} \end{array}$$

(9) $$\begin{array}{c} RU_{t}=C\text{Los}{\text{s}}_{t-1}+P\left(T_{t}\right)-\max P\left(T_{t},DB_{\text{End}}\right)\\ \text{if}\;MP_{t-1}=“\text{Short”,}\;t>0,\;\;T_{t}\neq DB_{\text{End}}. \end{array}$$

Otherwise, the value of *RU* is 0 under other circumstances.

The current *risk* is the maximal value among the current absolute values of *C*Loss, *DD*, *RU*, and the previous *risk*. The equation of risk is as follows:

(10) Risk  at  time  t:  Riskt =max⁡[|CLosst|,|DDt|,|RUt|,Risk(t−1)] if  t  >  0,Risk0=0 if  t=0.

#### 3.4.3. WinRate

The variable *WinRate* is the ratio between the number of complete trading orders (CTOs) with *net profit* > 0 and the total number of complete trading orders. The equation of *WinRate* is as follows:

(11) $$\begin{array}{c} \text{WinRate}=\frac{\left(\text{Total}\;\#\;\text{of}\;\text{CTOs}\;\text{with}\;NP>0\right)}{\left(\text{Total}\;\#\;\text{of}\;CTOs\right)}\times 100\%. \end{array}$$

#### 3.4.4. Profit Rules

The goal of this research is to discover all the profit rules in the TDB. Let *minProfit*, *maxRisk*, and *minWinRate* be user specified threshold values for the minimal profit, maximal risk, and minimal win rate, respectively. We define a profit rule to be a trading rule *R* with trading results [*Profit*_*R*_, *Risk*_*R*_, *WinRateR*] if *Profit*_*R*_  ≥  *minProfit*, *Risk*_*R*_ < *maxRisk*, and *WinRate*_*R*_  ≥  *minWinRate*, where *minProfit*  ∈  *R* ^*+*^, *maxRisk*  ∈  *R* ^*+*^, and *minWinRate*  ∈  *R* ^*+*^.

### 3.5. An Example of Trading Result

An example of trading rule {*BF*, *a*(0)*c*(−1), *b*(0)} is used to explain the profit rule mining in a trading simulation. The process is illustrated in Table 2.

Without loss of generality, we set *fee* to 1. At the beginning, the investor holds a close position HPOS_0_ = {*None*, 0,0} in the entry no. 0 of Table 2. The patterns *BP* = *a*(0)*c*(−1) and *SP* = *b*(0) occur at *TID* = 3 simultaneously causing a trading ambiguity; thus trading priority = “*BF*” is applied to solve the problem and generate the first trading order TO_1_ = {*Buy*, 1,500} in the entry no. 1 of Table 2. The trading order TO_1_ changes the close position to the long position HPOS_1_ = TO_1_ − HPOS_0_ = {*Buy*, 1,500}−{*None*, 0,0} = {*Long*, 1,500}. At this time *t* = 1, the investor holds a long position and expects the price of stock to go up. Since TO_1_ is not a CTO, no trading result is generated at time *t* = 1.

The next expected pattern is a SP at *TID* = 5 and *TO*_2_ = {*Sell*, 1, 490} in the entry no. 2 of Table 2 is generated to offset HPOS_1_{*Long*, 1, 500} to HPOS_2_ = {*Short*, 1, 490}. The calculation is HPOS_2_ = *TO*_2_ −  HPOS_1_ = {*Sell*, 1, 490} − {*Long*, 1, 500} = {*Short*, 1, 490}. At time *t* = 2, TO_1_ is a CTO because the HPOS_1_ is offset by *TO*_2_. After TO_1_ is changed to become a CTO, the trading result is generated. Since *MP*_1_ = *Long*, we have *NP*_1_ = 490 – 500 − 2 × 1 = −12, *C*Loss_1_ = *C*Loss_0_ + *NP*_1_ = 0 + (−12) = −12, *DD*_1_ = *C*Loss_0_ + minP(3, 5) − P(3) = 0 + 490 − 500 = −10, *RU*_1_ = 0, *Risk*_1_ = max(*|C*Loss_1_ *|*, *|DD*_1_ *|*, *|RU*_1_ *|*, *Risk*_0_) = max (*|*−12*|*, *|*−10*|*, *|*0*|*, 0) = 12, and the *WinRate*_1_ = (0/1) × 100% = 0% in the entry no. 1 of Table 2. At time *t* = 2, the investor holds a short position and expects the price to go down in the future.

The third expected pattern is a BP. Although BP = *a*(0)*c*(−1) and SP = *b*(0) occur at *TID* = 7 simultaneously, there is no trading ambiguity because current HPOS_2_ is not a close position and SP = *b*(0) is ignored. The BP at *TID* = 7 generates *TO*_3_ = {*Buy*, 1, 510} at the entry no. 3 of Table 2 and *TO*_3_ offsets HPOS_2_{*Short*, 1, 490} to HPOS_3_{*Long*, 1, 510}. HPOS_3_ is a long position which expects the price of stock to go up. *TO*_2_ is a CTO at time *t* = 3 such that *TO*_2_ has trading results. Because HPOS_2_ is a short position, we have *NP*_2_ = 490 − 510 − 2 × 1 = −22, *C*Loss_2_ = *C*Loss_1_ + *NP*_2_ = −12 + (−22) = −34, *DD*_2_ = 0, *RU*_2_ = *C*Loss_1_ + *P*(5) − max*P*(5, 7) = −12 + 490 − 510 = −32, *Risk*_2_ = max(*|*−34*|*, *|*0*|*, *|*−32*|*, 12) = 34, and *WinRate*_2_ = 0% at the entry no. 2 of Table 2.

The forth expected pattern is a SP at *TID* = 9 and *TO*_4_ = {*Sell*, 1, 570} is generated at the entry no. 4 of Table 2. *TO*_4_ offsets the HPOS_3_{*Long*, 1, 510} to HPOS_4_{*Short*, 1, 570}. Since *TO*_3_ is a CTO, we have trading results. Because HPOS_3_ is a long position, *NP*_3_ = 570 − 510 − 2 × 1 = 58, *C*Loss_3_ = *C*Loss_2_ + *NP*_3_ = −34 + 58 = 24 > 0; then *C*Loss_3_ is set to 0, *DD*_3_ = *C*Loss_2_ + *P*(7) − min*P*(7, 9) = −34 + 510 − 510= −34, *RU*_3_ = 0, *Risk*_3_ = max(*|*0*|*, *|*−34*|*, *|*0*|*, 34) = 34, and *WinRate*_3_ = (1/3) × 100% = 33% at the entry no. 3 of Table 2.

The last expected pattern is a BP, but there is no buy pattern BP coming after *TID* 9. However, it is not risk free because there are transactions from *TID* 9 to *DB*_End_ (i.e., *TID* 10). Because the last HPOS_4_ is a short position, we have *DD*_4_ = 0, *RU*_4_ = *C*Loss_3_ + *P*(9)−max*P*(9, 10) = 0 + 570 − 610 = −40, and *Risk*_4_ = max(*|*0*|*, *|*0*|*, *|*−40*|*, 34) = 40 at the entry no. 4 of Table 2.

Finally, the trading rule's *profit* = −12 + (−22) +58 = 24. Therefore, the trading result of the trading rule {*BF*, *a*(0)*c*(−1), *b*(0)} is [24, 40, 33%]. If *minProfit *≤ 24, *maxRisk* > 40, and *minWinRate *≤ 33%, then this trading rule is a profit rule.

## 4. Mining Profit Rules with Improved PRMiner

In this section, we review PRMiner and describe the improvements we made.

### 4.1. Terms and Definitions

We define a pattern *X* = *x*_1_(*i*_1_)*x*_2_(*i*_2_) ⋯ *x*_*j*_(*i*_*j*_), where *j* > 0 and 0 ≥*i*_*j*_≥ (1 − *maxspan*). Let shift(*X*, *k*) be a function to shift *X* into *X*′ and *X*′ = *x*_1_(*i*_1_ − *k*)*x*_2_(*i*_2_ − *k*) ⋯ *x*_*j*_(*i*_*j*_ − *k*), where *k* ≥ 0. For the pattern *a*(0) in Table 1, there exists a set of transaction IDs TIDSet(*a*(0)) = {3, 7}. After performing shift(*a*(0), 1), we have a new pattern *a*(−1) and TIDSet(*a*(−1)) = {4, 8}.

The number of items in a pattern is called the length of pattern. A pattern of length *k* is called *k*-pattern. For example, pattern *a*(0)*c*(0)*b*(−1) is a 3-pattern. Let *X* and *Y* be two patterns. We define a function join(*X*, *Y*) for joining these two patterns into a new pattern *Z*. For the pattern *Z* = join(*X*, *Y*), we have *Z* = *X* ∪ *Y* and TIDSet(*Z*) = TIDSet(*X*) ∩ TIDSet(*Y*). For example, the pattern *a*(0) appears in TIDSet(*a*(0)) = {3, 7} and the pattern *b*(0) can be found in TIDSet(*b*(0)) = {3, 5, 7, 9} in Table 1. Joining patterns *a*(0) and *b*(0), we have a new pattern *a*(0)*b*(0) whose TIDSet is {3, 7}.

Let *X* = *x*_1_(*i*_1_)*x*_2_(*i*_2_) ⋯ *x*_*m*_(*i*_*m*_) and *Y* = *y*_1_(*j*_1_)*y*_2_(*j*_2_) ⋯ *y*_*n*_(*j*_*n*_) be two patterns. We say that *X* = *Y*, if *x*_*K*_(*i*_*K*_) = *y*_*K*_(*j*_*K*_) for 1 ≤ *k* ≤ *m* = *n*, where *i*_1_ = *j*_1_ = 0. We say that *X* < *Y*, if *x*_1_(0) < *y*_1_(0) and there exists *k *≥ 1 such that *x*_*h*_(*i*_*h*_) = *y*_*h*_(*j*_*h*_) for 1 ≤ *h* ≤ *k* and *x*_(*k*+1)_(*i*_*(k*+1)_) < *y*_(k+1)_(*j*_(k+1)_). For example, *a*(0)*b*(0) < *a*(0)*b*(−1).

We define an ITSet that consists of a pattern of itemsets and a TIDSet. The form of ITSet is *x*_1_(*i*_1_)*x*_2_(*i*_2_) ⋯ *x*_*m*_(*i*_*m*_){*T*_1_, *T*_2_, …, *T*_*n*_}, where *T*_*n*_∈ *TID*. Let Ftid(*X*) be a function for getting the first TID of a pattern *X*. There exists an ambiguity if Ftid(*X*) = Ftid(*Y*) ≠ *ϕ*, where *Y* is a pattern. Let simulation (*R*) be a function using a trading rule *R* to do trading simulation and return the trading results *RS*_*R*_ where the form of *RS*_*R*_ is [*profit*_*R*_, *risk*_*R*_, *winrate*_*R*_].

Let *TP*′ = {*Both*, *BF*, *SF*} be a set of trading priorities for a trading rule. In PRMiner, we redefine the attribute trading priority *tp* ∈*TP*′ in a trading rule. A trading rule *R* with a trading priority “*Both*” means that one can extract two trading rules in which their trading priorities are “*BF*” and “*SF*”, respectively.

Let *X* and *Y* be two patterns. If *X* = *Y,* we can generate two trading rules with trading priority of *BF* and *SF*, respectively. If Ftid(*X*) *≠* Ftid(*Y*), we can generate two trading rules with the same trading priority = “*Both.*” If *X* *≠* *Y* and Ftid(*X*) = Ftid(*Y*), we can generate four trading rules as shown in Table 3, where all the combinations of rules for patterns *X* and *Y*are displayed.

Let RuleGenerator(*X*, *Y*) be a function for generating a set of trading rules by using patterns *X* and *Y* in a TDB. We say that an Mtr(*X*) is a set of megatransactions which contain the pattern *X*. The support *sup*(*X*) is defined as *|*Mtr(*X*)*|*. *X* is a frequent pattern if sup(*X*) ≥*minsup*, where *minsup* is a threshold value.

### 4.2. PRMiner and Its Improvements

Before mining profit rules, a user specifies the thresholds *minProfit*, *maxRisk*, and *minWinRate*. PRMiner assumes *minsup* = 1 if *minWinRate* > 0%; otherwise *minsup* = 0. It means that at least one occurrence of a profit rule is required for a positive *minWinRate*.

PRMiner scans a TDB only once and stores all of the 1-itemset into the *oneItemList* and the price is stored in the *PList*. The entry of the *oneItemList* contains an item *e* and its TIDset(*e*). The price for a specific TID *T*_*i*_ in *PList* is gotten by using the function *P*(*T*_*i*_) defined in Section 3.4.1.

Instead of a single genPattern, two new depth-first-search strategies of genBP and genSP are used in the improved PRMiner to generate BP and SP patterns, respectively, by joining and shifting the 1-pattern from *oneItemList* into *k*-pattern. Given the global parameters *maxspan* and *minsup*, Pseudocodes 1 and 2 show the pseudocode of genBP and genSP, respectively. To terminate the algorithm, the test of checking if the depth of search = 0 is replaced by examining the end of genBP and genSP in their respective procedures for the purpose of better performance.

The genBP and genSP procedures are based on the trading principles and the functions defined in the last section. First, the improved PRMiner calls genBP to generate a BP pattern. Then, genSP is invoked to produce all SP patterns using the BP as one of its input parameters. During the construction of SP patterns, the trading rules are generated by calling RuleGenerator as shown in Line 16 of Pseudocode 2 under the condition BP ≤ SP. After generating the second BP pattern, genSP is invoked again to produce corresponding SP patterns and trading rules as before. The process continues until the end of genBP is reached. Without rescanning the TDB, these trading rules are used to do trading simulation and produce trading results. Instead of providing an intricate description, we use a simple example in the next section to illustrate the mining process of the improved PRMiner.

### 4.3. An Example of Using the Improved PRMiner

**Figure 4:**
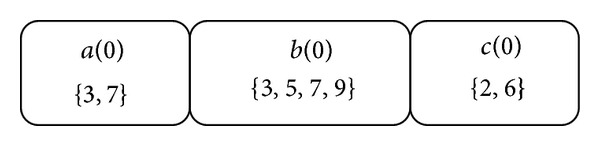
The oneItemList for Table 1.

We use the TDB of Table 1 and set the mining parameters *fee* = 1, *maxspan* = 2, *minProfit* = 20, *maxRisk* = 50, and *minWinRate* = 30%. At the beginning, the TDB is scanned to get the oneItemList as shown in Figure 4 where each entry contains an item *e* and TIDSet(*e*). As described in Section 4.2, the *minsup* is set to 1 since *minWinRate* > 0%.

The improved PRMiner uses the functions genBP and genSP to generate BP and SP patterns as shown in Tables 10 and 11, respectively. The first three BP patterns generated by the improved PRMiner in Table 10 are *a*(0){3,7}, *a*(0)*b*(0){3,7}, and *a*(0)*b*(0)*c*(0){}. Since the third BP has an empty TIDSet with a support value of zero, the pattern *a*(0)*b*(0)*c*(0) is removed and all of its extended patterns *a*(0)*b*(0)*c*(0)*a*(−1), *a*(0)*b*(0)*c*(0)*a*(−1)*b*(−1), and *a*(0)*b*(0)*c*(0)*a*(−1)*b*(−1)*c*(−1) are not generated. Similarly, the fourth BP *a*(0)*b*(0)*a*(−1) and other patterns with an empty TIDSet are processed in the same manner. This filtering process makes the improved PRMiner more efficient.

After a new pattern BP is generated by genBP, genBP calls genSP to generate SP patterns and invokes RuleGenerator to produce trading rules using the pair of BP and SP patterns if the condition BP ≤ SP is satisfied. For example, RuleGenerator uses the first BP *a*(0) from the *seq*. 1 of Table 10 and SP *a*(0) from the *seq*. 1 of Table 11 to form two rules {*BF*, *a*(0), *a*(0)} and {*SF*, *a*(0), *a*(0)}. However, the first BP *a*(0) and the second SP *a*(0)*b*(0) cannot be used to generate trading rules because *a*(0) > *a*(0)*b*(0). Therefore, SP *a*(0)*b*(0) and its extended pattern SP *a*(0)*b*(0)* c*(0) are ignored. The patterns of *seq.* 3~6 in Table 11 are all ignored because their support value is zero.

As for the first BP *a*(0) and the SP* b*(0) of *seq*. 7 in Table 11, four trading rules are generated. They are {*BF*, *a*(0), *b*(0)}, {*SF*, *a*(0), *b*(0)}, {*BF*, *b*(0), *a*(0)}, and {*SF*, *b*(0), *a*(0)}. It is a simple combination of values for the components of a trading rule based on the rules in Table 3. After all the trading rules are generated by using BP *a*(0) and the rest of SPs, the next BP is changed to the second BP *a*(0)*b*(0) of *seq*. 2 in Table 10 and genBP calls genSP again to generate SPs and associated trading rules. The process continues until the end of genBP is reached.

## 5. Experiments

We use Java with JDK 1.6 to implement PRMiner and improved version. Our computer has a Intel Core-2 Duo P9600 CPU @ 2.66 GHz and 4 GB RAM. The operating system is Windows XP. All discovered profit rules are counted to compute the statistics and are not stored into the disk.

Two types of experiments were performed to evaluate the performance of the improved PRMiner. In the first type, we used a real dataset to mine profit rules and compared the result with that of the famous Buy and Hold Strategy (BHS) [13] to show the effectiveness of our algorithm.  Section 5.1 describes the preprocessing for the real world dataset. A simple and effective measurement, Return of Investment (ROI) [14], is employed as the criteria for effectiveness evaluation in Section 5.2. Next, synthetic datasets were generated by using the IBM Data Generator [15] to measure the run time efficiency of our improved PRMiner using various minProfit, *maxRisk*, and *minWinRate* values in Section 5.3.

### 5.1. Data Preprocessing

The real dataset comes from the Taiwan Economic Journal [16]. We select the data of the futures market in Taiwan from 2005/1/3 to 2010/6/30. Our approach is applicable to stock markets too. The source dataset contains 35,602,531 transactions of tick data including the date, time, price, and volume information. The processed tick data are stored in a TDB with the format of Table 1. The following three preprocessing steps are required to make the final conversion.

1. Convert the tick data into candlestick chart (a style of bar chart) [17].
2. Use indicator functions to generate indicators on the candlestick chart.
3. Transform the candlestick chart and indicators into the format of transactional DB.

First, the tick data are converted into candlestick chart as shown in Table 4. Each transaction has three attributes of date, time, and prices representing the trading at that time. After conversion each bar in the candlestick includes six attributes of date, time, open price, highest price, lowest price, and close price. Each of the bars merges the price of tick data according to a specific time period *K* (*K* > 0). A *K*-minute (or *K*-min.) candlestick chart consists of the bars within a *K*-minute time period. For example, the transactions of one day in the tick data are partitioned into *n* periods of *K*-minute and a bar is generated in the candlestick chart for each period of the tick data. The prices of the first and last transactions in a period correspond to the open and close prices of a bar, respectively. The highest and lowest prices in a period also correspond to the highest and lowest prices in a bar, respectively.

Second, technical indicators are used on the candlestick chart for technical analysis of financial market data. TA-Lib [18] provides libraries for generating useful indicators. One of the simple and widely used indicators is a simple moving average (SMA). We chose the function of SMA(*b*) to generate the average value of close prices among *b* bars and return a numerical value for each bar, where *b* is an integral value. For example, function SMA(3) calculates the average value of three close prices starting from the current bar and return a numerical value. For example, the SMA(3) at time = “905” (the third entry of Table 4) is (6177 + 6177 + 6182)/3 = 6179 and SMA(3) at time = “915” (the fourth entry of Table 4) is (6177 + 6182 + 6165)/3 = 6175. Based on our experiments, we chose three indicators SMA(3), SMA(5), and SMA(8) to generate three numerical values for each bar.

Third, the candlestick chart along with indicators is converted into the form that can be stored as a transaction in our TDB. Each bar corresponds sequentially to one transaction in the TDB. The TID value in the TDB is a series number starting from 1. The close price in a bar is the price of a transaction. To generate items for each transaction, we define two functions cross-over and cross-under to work on the indicators.

**Figure 5:**
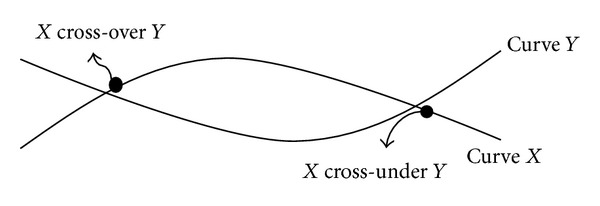
Diagram for cross-over and cross-under functions.

Figure 5 shows the functions of cross-over and cross-under. The horizontal axis is time and the vertical axis is close price. Two curves *X* and *Y* are shown with two cross-points in the Figure 5. At the cross-points, the prices of *X* and *Y* are the same. After passing the left cross-point on curve *X*, the price of curve *X* is greater than the price of curve *Y* and it is called a cross-over event. After passing the right cross-point on curve *X*, the price of curve *X* is smaller than the price of curve *Y* and it is called a cross-under event. Three indicators SMA(3), SMA(5), and SMA(8) are used with the cross-over and cross-under functions to generate six events which are represented by items *a* to *f* as shown in Table 5.

### 5.2. Effectiveness of Profit Rules

An important benchmark, called Buy and Hold Strategy (BHS), is used to evaluate the effectiveness of our profit rules. BHS means that an investor buys a stock at the first transaction and does not sell the stock until the last transaction. The profit of BHS is the price at the last transaction minus the price at the first transaction while the risk of BHS is the price at the first transaction minus the lowest price in the TDB.

The return of investment (ROI) [18] is a simple and effective measurement for profitability and the definition of ROI is as follows:

(12) $$\begin{array}{c} \text{ROI}=\frac{\text{Profit}}{\text{Cost}\_\text{of}\_\text{Investment}}. \end{array}$$

Here “profit” is the profit of a trading rule. The cost of investment is the summation of risk and initial margin, where the initial margin in the futures market is the amount of cash an investor must put up to open an account for trading. The investor must have enough capital to cover the risk during the investment period. The higher value of ROI indicates the better profitability of a trading rule.

In the first type of evaluation we use the real world dataset to perform two sets of experiments to measure the effectiveness of our improved PRMiner. The first set of experiments is to compare our results with that of BHS. The second set further evaluates the effectiveness of the mining algorithm by adjusting the values of minProfit, *maxRisk*, and *minWinRate* parameters.

We set *fee* = 4, *minProfit* = 982, *maxRisk* = 2290, and *minWinRate* = 30%, *maxspan* = 2, and the initial margin of Taiwan trading market = 320. The results of the first set of experiments are shown in Table 6 where the second column displays the ROI for BHS and the last three columns are for our improved PRMiner with the maximal, average, and minimal ROIs. Five different *K*-minute time periods of candlestick data were used in the experiments where *K* = 10, 15, 20, 30, and 60. It is obvious to see that all the ROI values of the improved PRMiner are much better than those of BHS. In particular, the second best Min. ROI of the improved PRMiner is greater than the best ROI of BHS (i.e., 0.39 > 0.38) in the last row of Table 6. Here, the best result of the improved PRMiner comes from the 30-minute candlestick data with the Max. ROI = 16.71, Avg. ROI = 2.56, and Min. ROI = 0.38. All of them are better than BHS's ROI = 0.36.

In the second set of experiments we use 10-Min. candlestick data to work with different settings of minProfit, *minWinRate*, and *MaxRisk*. We set fee = 4 and maxspan = 2. Users can set their expected value of minProfit. Here we assume minProfit = 4000.

To find the suitable settings of *minWinRate* for better ROIs, we first specify the *minWinRate* values without using the *maxRisk* (i.e., *maxRisk* is set to a very large value like 999999) in the first experiment. The results are shown in Table 7. As the *minWinRate* increases from 60% to 100%, the Min. ROI becomes larger in a linear fashion as expected. The Avg. ROI has a similar trend except when the *minWinRate* goes up to 90% and 100% since there are fewer profit rules to work with as shown in the last two columns of Table 7. Regarding the Max. ROI, the largest one is 16.01 when the *minWinRate* is below 90%. After that the Max. ROI becomes 9.88 since the previous rules may not satisfy the new requirement.

Second, we set the range of *maxRisk* from 3000 down to 1000 without specifying the *minWinRate* (i.e., *minWinRate* = 0%).  Table 8 shows the results where the Avg. ROI and Min. ROI increase in response to lower risks. In the third experiment we specify both of *minWinRate* and *maxRisk*. To illustrate how to find better ROIs, we select the *maxRisk* that produces the best ROI (i.e., *maxRisk* = 1000) and set the range of *minWinRate* from 60% to 100%. As a result, except that the Max. ROIs remain the same (i.e., 16.01 and 9.88), all the other values of the Avg. ROIs and Min. ROIs in Table 9 are better than those in Table 7. From these experiments we can observe that the profit rules in our real world example are a small subset and can be extracted effectively by using profit-mining algorithms to get good ROIs. As expected, the more parameters of minProfit, *minWinRate*, and *maxRisk* are specified the better ROIs one can get. In particular, the increase rates of Min. ROIs from Table 7 to Table 8 and from Table 8 to Table 9 are twofold.

### 5.3. Performance Evaluation

We designed two sets of experiments to evaluate the execution performance of the improved PRMiner. By varying three user specified parameters of minProfit, *maxRisk*, and *minWinRate* in the first set of experiment, we can observe the changes of performance in executing the improved PRMiner. Stable performance is a very important feature for a financial tool. In the second set of experiments, we increase the number of transactions to test if the improved PRMiner is a scalable algorithm. The results are plotted on the diagrams using the average value of ten tests.

We used the IBM data generator to build test datasets For example, the first dataset is T3I2D20KN4, where *T* = 3 is the average size of transactions, *I* = 2 is the average size of maximal potentially large itemsets, *D* = 20 K is the total number of transactions, and *N* = 4 is the number of unique items. In addition, a price generator was created to randomly produce the price information for each transaction in order to have the same format of Table 1. Given a seed of 1000 as the base price, we set that the range of the maximal and minimal prices between two consecutive transactions is within 2%, that is, between 1020 and 980. There are also ceilings for the highest and lowest prices in the TDB.

As before, we set fee = 1 and maxspan = 2. In the first set of experiments, we fix two of the three threshold parameters and change the values of the third one in a specific range.

**Figure 6:**
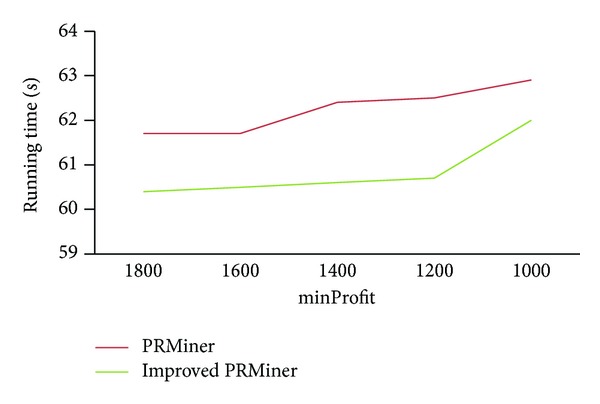
Performance comparison for various minProfit values.

First, we set *maxRisk* = 800 and *minWinRate* = 50%.  Figure 6 shows the changes of running time for the values of minProfit ranging from 1000 to 1800.

**Figure 7:**
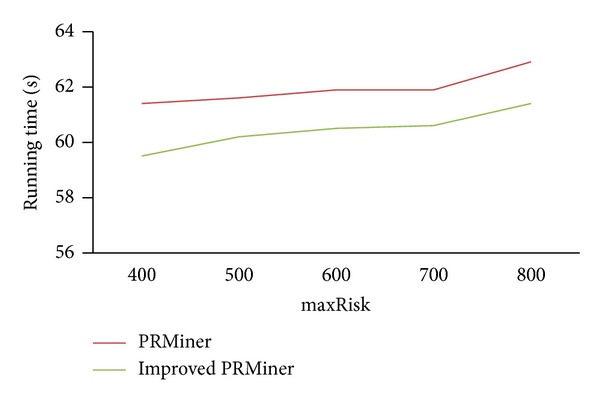
Performance comparison for various *maxRisk* values.

Similarly, Figure 7 shows the changes of running time for the settings of minProfit = 1000, *minWinRate* = 50%, and *maxRisk* = 400~800.

**Figure 8:**
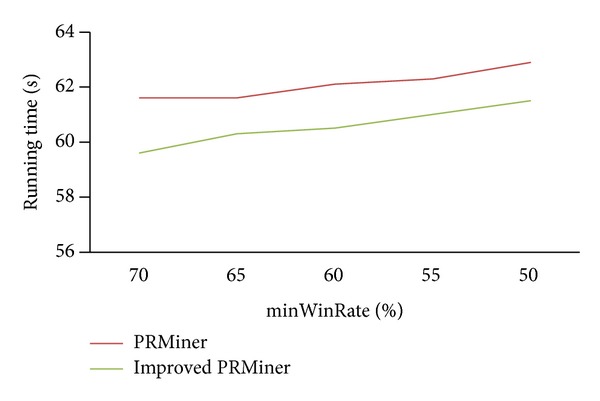
Performance comparison for various minWinRates.

Finally, Figure 8 displays the changes of running time for the settings of minProfit = 1000, *maxRisk* = 800, and *minWinRate* = 50%~70%.

From the above three experiments, we can see the range of running time is between 59.5 and 62 seconds. These results demonstrated that the improved PRMiner performs better than the PRMiner and both are stable and scalable.

**Figure 9:**
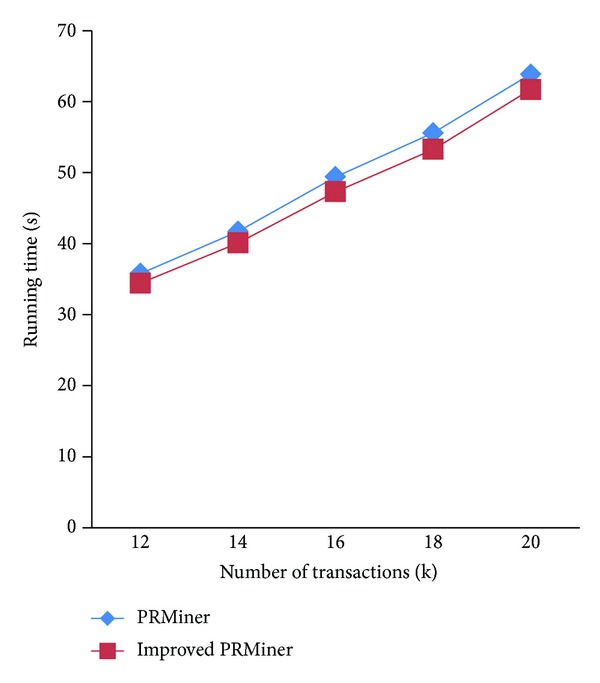
Performance comparison for different TDB sizes.

In the next set of experiments, we focus on the performance of the improved PRMiner for five databases of different sizes. The thresholds are maxspan = 2, fee = 1, minProfit = 1000, *maxRisk* = 800, and *minWinRate* = 50%.  Figure 9 shows that the performance of the improved PRMiner scales up almost linearly with respect to the increase of the number of transactions from 12 K to 20 K in the TDB. Although the overall performance of the improved PRMiner is better than that of the original PRMiner, there are cases where the improvements (e.g., at 14 K and 18 K) are slightly less than the others.

**Figure 10:**
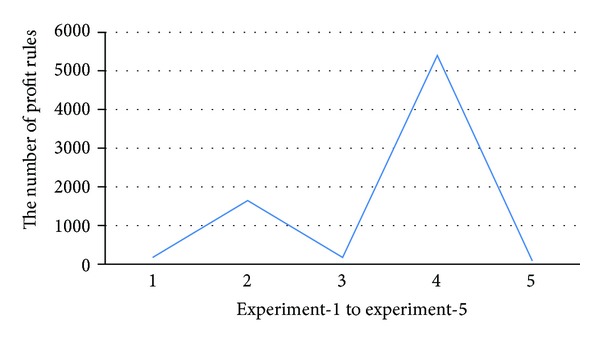
The numbers of profit-rules from five different price distributions.

**Figure 11:**
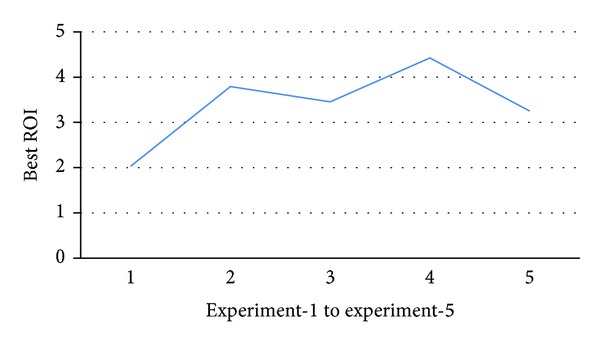
The best ROIs from five different price distributions.

To find the cause of degradation, we set the size of TDB = 14 K and repeat the last experiment five times with different distributions of prices generated randomly from the price generator. From Figure 10, we can see the large variation on the number of generated profit rules where the numbers of profit rules are close to zero in three out of five times. Therefore, it explains why the improvement is not as much as the others since there are less number of rules. Additionally, Figure 11 shows that the variation of price distributions and the number of generated profit rules do not affect the best ROI. The results reinforce that the improved PRMiner is scalable and stable.

## 6. Conclusion and Future Work

In this paper, we improved the PRMiner algorithm and its implementation for effective mining of profit rules. Many examples are used to show how to mine profit rules and obtain good trading results. Experimental results show the effectiveness of our approach and its quality features on scalability and stable performance. As a preliminary study on financial data mining, we only used a simple trading approach of interday model for trading simulation. Using different trading models, we can derive many different trading rules under specific transaction databases. In data mining research, one expects to mine any knowledge that users might be interested in. These useful results of knowledge can be discovered and presented in terms of rules, patterns, or any other forms to meet users' expectation. Similarly, in profit-mining, we think more types of profit rules can be defined and discovered in trading data from any kind of financial sectors. Since investors have their own measuring criteria for mining the preferred trading rules, we will investigate more useful models of financial data mining in the future. There is also room to improve the performance of our profit-mining algorithms.

## Figures and Tables

**Table 1:** An example of transactional database (TDB).

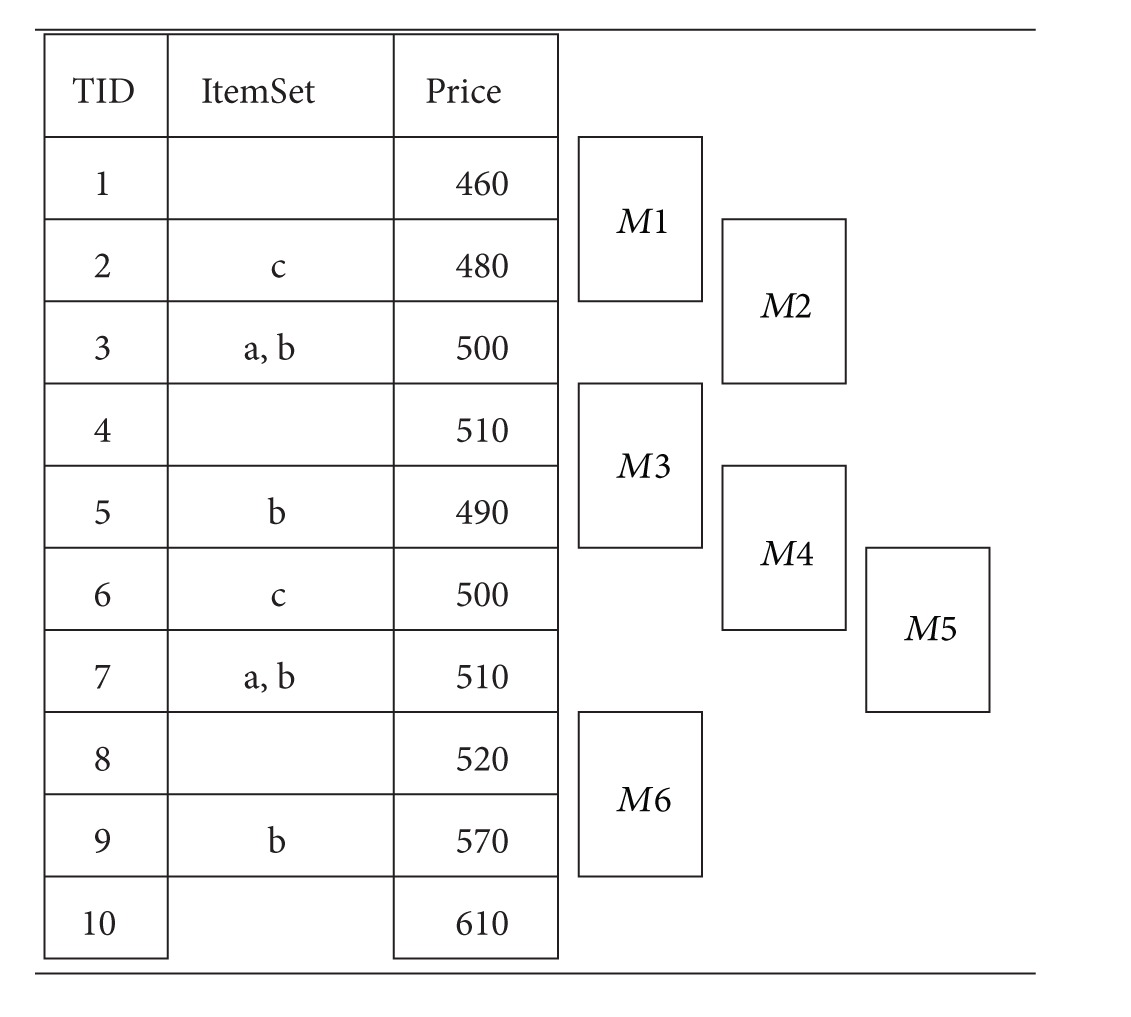

**Table 2:** The simulated trading of the rule {BF, *a*(0)*c*(−1), *b*(0)}.

No	Trading order			TID	Hold position			NP	CLoss	DD	RU	Trading result		
	*tc *	*qty *	*price *		*mp *	*hqty *	*hprice *					Profit	Risk	WinRate
0					None	0	0	0	0	0	0	0	0	0%
1	Buy	1	500	3	Long	1	500	−12	−12	−10	0	−12	12	0%
2	Sell	1	490	5	Short	1	490	−22	−34	0	−32	−34	34	0%
3	Buy	1	510	7	Long	1	510	58	0	−34	0	24	34	33%
4	Sell	1	570	9	Short	1	570			0	−40		40	

**Table 3:** The combination of trading rules using patterns X and *Y*.

Conditions	The combination of trading rules
Ftid(*X*) *≠* Ftid(*Y*)	Rule 1: {Both, *X*, *Y*}
	Rule 2: {Both, *Y*, *X*}

*X* = *Y*	Rule 3: {BF, *X*, *Y*}
	Rule 4: {SF, *X*, *Y*}

*X* ≠ *Y* and Ftid(*X*) = Ftid(*Y*)	Rule 5: {BF, *X*, *Y*}
	Rule 6: {SF, *X*, *Y*}
	Rule 7: {BF, *Y*, *X*}
	Rule 8: {SF, *Y*, *X*}

**Table 4:** 10-minute candlestick chart and SMA indicators.

Date	Time	Open	Highest	Lowest	Close	SMA(3)	SMA(5)
20050103	845	6185	6193	6176	6177	0	0
20050103	855	6178	6185	6173	6177	0	0
20050103	905	6178	6186	6175	6182	6179	0
20050103	915	6182	6189	6163	6165	6175	0
20050103	925	6167	6179	6164	6170	6172	6174
20050103	935	6169	6171	6154	6164	6166	6172
20050103	945	6166	6173	6162	6173	6169	6171
20050103	955	6173	6176	6170	6173	6170	6169
20050103	1005	6173	6176	6172	6173	6173	6171

**Table 5:** The items of generated events in the experiment.

Item	Event
*a*	SMA(3) cross over SMA(5)
*b*	SMA(3) cross down SMA(5)
*c*	SMA(3) cross over SMA(8)
*d*	SMA(3) cross down SMA(8)
*e*	SMA(5) cross over SMA(8)
*f*	SMA(5) cross down SMA(8)

**Table 6:** ROI comparison between BHS and the improved PRMiner.

CandleStick	ROI for BHS	Max. ROI	Avg. ROI	Min. ROI
10 Min.	0.36	16.01	2.21	0.38
15 Min.	0.36	12.12	1.94	0.38
20 Min.	0.36	8.46	2.24	0.41
30 Min.	0.36	16.71	2.56	0.38
60 Min.	0.38	12.39	2.37	0.39

**Table 7:** Mining results of using various minWinRates.

maxRisk	minWinRate	Max. ROI	Avg. ROI	Min. ROI	No. of profit rules	No. of profit rules/no. of total rules
999999	60%	16.01	2.85	0.67	4027	0.0124%
999999	70%	16.01	3.01	0.76	1639	0.0050%
999999	80%	16.01	3.59	0.78	807	0.0025%
999999	90%	9.88	3.04	0.82	166	0.0005%
999999	100%	9.88	3.2	1.06	154	0.0005%

**Table 8:** Mining results of using various maxRisks.

maxRisk	minWinRate	Max. ROI	Avg. ROI	Min. ROI	No. of Profit Rules	No. of profit rules/no. of total rules
3000	0%	16.01	3.02	1.22	6700	0.0206%
2500	0%	16.01	3.45	1.43	4904	0.0151%
2000	0%	16.01	3.91	1.79	3289	0.0101%
1500	0%	16.01	4.47	2.52	1949	0.0060%
1000	0%	16.01	5.12	3.32	670	0.0021%

**Table 9:** Mining results of using various minWinRates with maxRisk = 1000.

maxRisk	minWinRate	Max. ROI	Avg. ROI	Min. ROI	No. of Profit Rules	No. of profit rules/no. of total rules
1000	60%	16.01	5.53	3.32	480	0.00148%
1000	70%	16.01	8.5	3.32	140	0.00043%
1000	80%	16.01	8.69	4.24	120	0.00037%
1000	90%	9.88	8.39	6.02	22	0.00007%
1000	100%	9.88	8.39	6.02	22	0.00007%

**Table 10:** The sequence of generated patterns using genBP.

Seq.	Pattern	TIDSet
1	*a*(0)	{3,7}
2	*a*(0)*b*(0)	{3,7}
3	*a*(0)*b*(0)*c*(0)	{}
4	*a*(0)*b*(0)*a*(−1)	{}
5	*a*(0)*b*(0)*b*(−1)	{}
6	*a*(0)*b*(0)*c*(−1)	{3,7}
7	*a*(0)*c*(0)	{}
8	*a*(0)*a*(−1)	{}
9	*a*(0)*b*(−1)	{}
10	*a*(0)*c*(−1)	{3,7}
11	*b*(0)	{3,5, 7,9}
12	*b*(0)*c*(0)	{}
13	*b*(0)*a*(−1)	{}
14	*b*(0)*b*(−1)	{}
15	*b*(0)*c*(−1)	{3,7}
16	*c*(0)	{2,6}
17	*c*(0)*a*(−1)	{}
18	*c*(0)*b*(−1)	{6}
19	*c*(0)*b*(−1)*c*(−1)	{}
20	*c*(0)*c*(−1)	{}

**Table 11:** The sequence of generated patterns using genSP.

Seq.	Pattern	TIDSet
1	*a*(0)	{3,7}
2	*a*(0)*b*(0)	{3,7}
3	*a*(0)*c*(0)	{}
4	*a*(0)*a*(−1)	{}
5	*a*(0)*b*(−1)	{}
6	*a*(0)*c*(−1)	{}
7	*b*(0)	{3,5, 7,9}
8	*b*(0)*c*(0)	{}
9	*b*(0)*a*(−1)	{}
10	*b*(0)*b*(−1)	{}
11	*b*(0)*c*(−1)	{3,7}
12	*c*(0)	{2,6}
13	*c*(0)*a*(−1)	{}
14	*c*(0)*b*(−1)	{6}
15	*c*(0)*b*(−1)*c*(−1)	{}
16	*c*(0)*c*(−1)	{}
